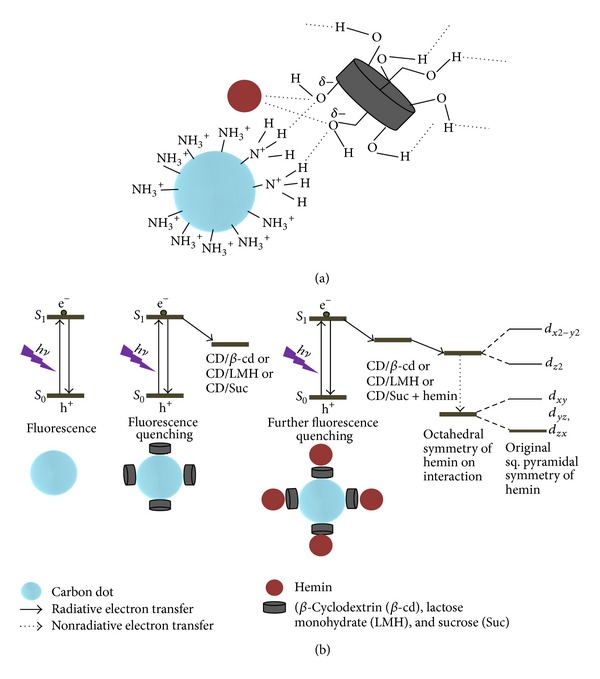# Capped Fluorescent Carbon Dots for Detection of Hemin: Role of Number of –OH Groups of Capping Agent in Fluorescence Quenching

**DOI:** 10.1155/2013/529159

**Published:** 2013-12-28

**Authors:** Upama Baruah, Neelam Gogoi, Gitanjali Majumdar, Devasish Chowdhury

**Affiliations:** ^1^Physical Sciences Division, Polymer Unit, Institute of Advanced Study in Science and Technology, Paschim Boragaon, Garchuk, Guwahati 781035, India; ^2^Department of Chemistry, Assam Engineering College, Jalukbari, Guwahati 781013, India

## Abstract

We have successfully demonstrated the use of capped carbon dot systems, namely, CDs/**β**-cd, CDs/LMH, and CDs/Suc, as fluorescent sensors for the detection of hemin. The capped carbon dot systems showed quenching of PL intensity in the presence of hemin. The minimum detection limit was determined to be ~1 **μ**M. The PL response with free Fe(II) and Fe(III) was also studied. It was observed that PL quenching of capped carbon dot systems in the presence of hemin is dependent on the number of –OH groups in the capping agent. The order of quenching towards hemin was determined to be CDs/**β**-cd > CDs/LMH = CDs/Suc > CDs. A possible mechanism to account for the observation is also discussed in the paper.

## 1. Introduction

In the recent years carbon based nanomaterials have attracted particular attention due to their unique structural and physical properties. Sp^2^ bonded graphitic carbon is found in all reduced dimensionalities including zero-dimensional fullerenes [1], one-dimensional carbon nanotubes (CNTs) [2–4], and two-dimensional graphene [5–7]. On the other hand very recently, a new member has been added to this family, namely, photoluminescent carbon dots (CDs) which are sp^3^ [8–10] hybridised. This new class of materials has drawn considerable attention due to its unique properties and applications. The characteristic properties of carbon dots include (i) their unique small size (3–10 nm), (ii) the ease of passivation of their particle surface by organic or other molecules via covalent linkages or by chemical adsorption, (iii) their ready solubility in water, (iv) their characteristic bright blue or green fluorescence under irradiation with UV light [11], (v) biocompatibility, and (vi) good photostability [12]. The gradually increasing attraction towards carbon dots may be attributed to availability of source, resistance to photobleaching, inexpensive nature, and strong and tunable photoluminescence. Since photoluminescence from carbon dots can be quenched efficiently by either electron acceptor or electron donor molecules, it is indicated that photoexcited carbon dots are excellent electron donors and acceptors [13]. This may be attributed to passivated defects on carbon particle surface acting as activation energy gap [10]. All these advantages of carbon dots make them promising candidates for applications in optoelectronic devices, for fluorescent biosensing or imaging [12], biomedical labeling, drug delivery and in the field of photocatalysis [8]. With recent advances in technology, an increasing number of C-dots based applications have been carried out, such as cell imaging, dye degradation, pH determination, chemiluminescence, and immunoassay. Zhou and co-workers [8] have found that C-dots prepared by pyrolysis of ethylenediamine- tetraacetic acid (EDTA) salts can be used as a novel fluorescence turn off and on based biosensor for Hg^2+^ and biothiols. Zhou et al. [8] reported the potential of C-dots passivated with PPEI-EI for two-photon luminescence microscopy bioimaging using human breast cancer MCF-7 cells. Another significant advantage of using carbon dots is that they can be capped with some other organic molecules which may lead to tuning of their intensity of photoluminescence [14] and this may be attributed to passivated defects on carbon particle surface acting as activation energy gap. This property can be employed for designing carbon dots based sensors for detection of various analytes.

The importance of heme in biological and environmental systems has eventually led to recent interest in the development of selective techniques for both qualitative and quantitative determination of iron in complex protein systems. Although various analytical techniques such as spectrophotometry, inductively coupled plasma mass spectrometry, voltammetry, and atomic absorption spectroscopy have been used for sensitive iron determination, they require complicated pretreatment procedures and the use of sophisticated instrumentation. Moreover, they suffer from interference by other ions. Thus, in the recent years, fluorescent sensors have been widely investigated for the selective detection of heme because of their ability to provide a simple, sensitive, selective, precise, and economical method for monitoring up to very concentrations of target metal ions without any pretreatment of the sample [15]. Many fluorescence based sensors have been designed till date for selective and sensitive detection of heme. Sharon et al. have developed a new phenomenon, where the G-quadruplex/hemin structure associated with CdSe/ZnS QDs quenches the luminescence of the nanoparticles. This enabled the development of new optical sensing platforms for the detection of DNA and for the analysis of aptamer-substrate complexes [16]. Qu et al. have demonstrated a new type of CNP based sensor for label-free detection of Fe^3+^ and dopamine with high sensitivity and selectivity [17]. Han et al. have developed a facile and feasible method for discriminating and quantifying heme (Fe(II)) and hemin (Fe(III)) by employing aqueous CdTe QDs linked with different surface ligands, such as thioglycolic acid TGA, 3-mercaptopropionic acid MPA, and 1-thioglycerol TG [18].

In the present work we have designed a novel system based on fluorescent carbon dots capped with *β*-cyclodextrin, lactose monohydrate, and sucrose for sensitive and selective detection of iron ions in aqueous media both in porphyrin system, hemin in this case, and in the free state. As hemin is a salt, containing a chloride ligand in the fifth coordination site of Fe(III) in place of histidine in the real protein, it can easily be used as a model system for heme proteins. Capping of carbon dots with *β*-cyclodextrin has already been reported by our group showing its use for the detection of fluoride ions [19]. In this paper we have tried to elucidate the role of the capping agent used on the photoluminescence (PL) properties of the system. Iron(III) being a hard Lewis acid can interact with hard Lewis bases like hydroxyl groups. Based on this we have chosen the capping agents bearing only hydroxyl groups. Interestingly we have found that the number of hydroxyl groups in the capping agent has direct bearing on the PL properties (quenching) used for detection of Fe(III) in hemin. Therefore, this system not only provides a novel design for highly sensitive detection of iron(III) in hemin with a detection limit as low as 1 *μ*M but also has advantages of low toxicity compared to heavy metal based quantum dots.

## 2. Experimental

### 2.1. Materials

Chitosan (low molecular weight), *β*-cyclodextrin (*β*-cd), and hemin were purchased from Sigma Aldrich. Glycerol (98% purified), sodium hydroxide NaOH, lactose monohydrate, sucrose GR, iron(II) sulphate heptahydrate GR FeSO_4_, and iron(III) chloride anhydrous FeCl_3_ were purchased from Merck. Glacial acetic acid (99.5%) used was purchased from Qualigens. The water used was from a Milli-Q water purification system.

### 2.2. Synthesis of Carbon Dots (CDs) from Chitosan Hydrogel

Chitosan hydrogel was synthesized as reported earlier by our group [9]. Briefly, 0.1 g of chitosan was taken in a beaker and 10 mL of a 1 : 3 mixture of 1% glacial acetic acid and glycerol was added to it and stirred with the help of a magnetic stirrer for 2 hours at room temperature. To the resulting clear solution 300 *μ*L of 5 N NaOH was added. Immediately a clear and slightly tacky hydrogel was formed. This hydrogel was then washed with plenty of water and stored in water.

Carbon Dots (CDs) from chitosan hydrogel were prepared by taking a small amount of the prepared hydrogel and dissolving it in 20 mL 0.1 M acetic acid and the resulting solution was microwaved for 20 minutes. The formation of CDs was confirmed by the appearance of a bluish glow when placed under UV light.

### 2.3. Capping of the Prepared CDs with *β*-Cyclodextrin (*β*-cd), Lactose Monohydrate (LMH), and Sucrose (Suc)

For the capping of CDs, 400 *μ*L of 0.5 mM *β*-cyclodextrin solution or lactose monohydrate or sucrose in water was added dropwise to 2 mL of the prepared CDs solution with continuous stirring for 2 hours at room temperature. The CDs/*β*-cd or CDs/LMH or CDs/Suc thus obtained was characterized by PL and dynamic light scattering (DLS) techniques.

### 2.4. Detection of Hemin by CDs/*β*-cd, CDs/LMH, and CDs/Suc

The hemin detection was monitored using a spectrofluorometer. The capped CDs, that is, CDs/*β*-cd or CDs/LMH or CDs/Suc, were taken in a quartz cuvette and 2 mM solution of hemin was added in amount of 10 *μ*L for each sampling and after 1 min of time gap the corresponding fluorescence spectra were recorded. Detection of free Fe(III) in FeCl_3_ and Fe(II) in FeSO_4_ was also done in a similar way.

## 3. Results and Discussion

**Figure 1:**
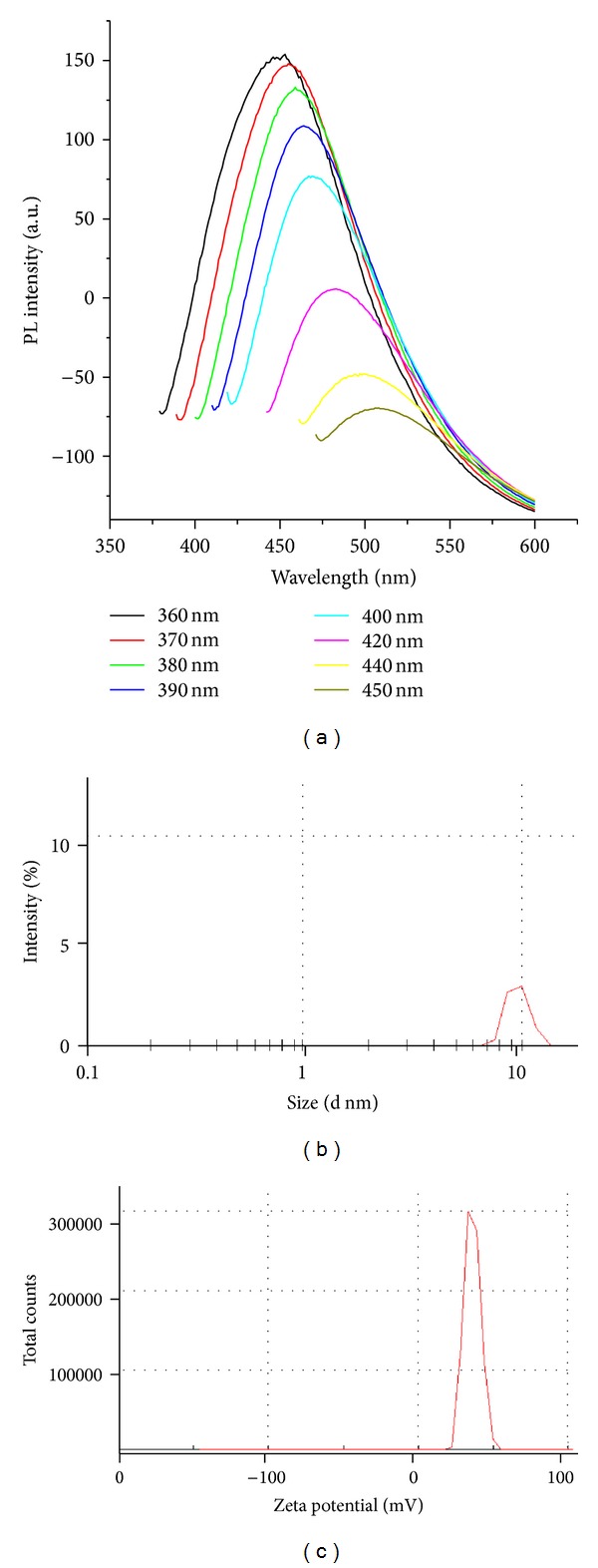
(a) The stacked photoluminescence (PL) spectra of CDs excited at different wavelengths (from 370 nm to 450 nm), (b) the particle size distribution graph of CDs (average size ~9.7 nm), and (c) zeta potential graph of CDs (ZP = 36.2 mV).

**Figure 2:**
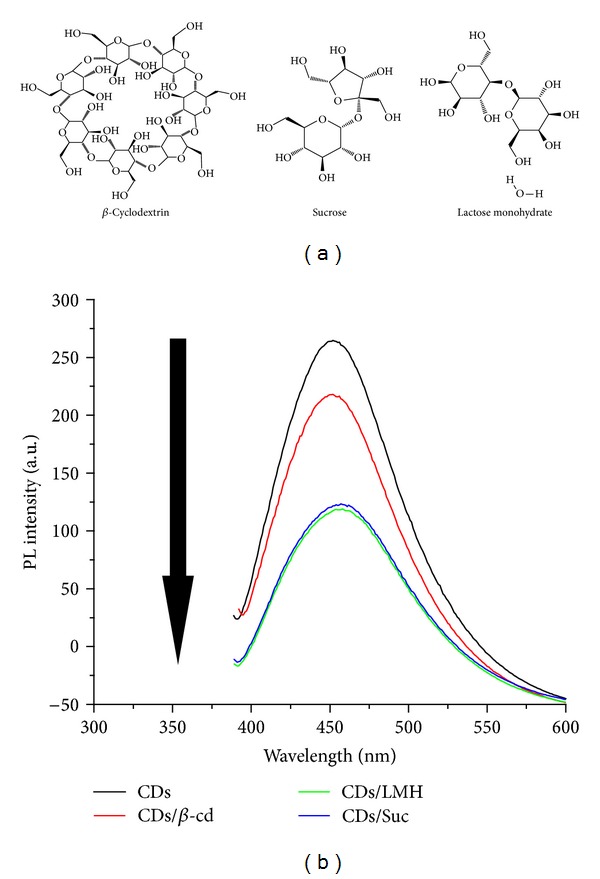
The photoluminescence (PL) spectra of CDs capped with *β*-cyclodextrin (*β*-cd), lactose monohydrate (LMH) and sucrose (Suc), and excited at 370 nm.

Carbon dots (CDs) were prepared from chitosan hydrogel by the method developed and reported earlier by our laboratory. CDs being highly fluorescent materials, photoluminescence properties were investigated.  Figure 1(a) shows the photoluminescence (PL) spectra of CDs excited at different wavelengths. It was observed that with increase in the excitation wavelength from 370 nm to 450 nm, the emission from CDs is gradually shifted, accompanied by decreased fluorescence intensity. This shift of emission from CDs has also been reported previously by us and other groups. It was observed that excitation at 360, 370, 380, 390, 400, 420, 420, 440, and 450 nm resulted in emission at 453, 455, 459, 464, 469, 483, 499, and 505 nm, respectively. As far as the PL mechanism of CDs is concerned it is believed that both the quantum size effect and surface defects contribute to PL. The particle size of CDs was determined by dynamic light scattering (DLS) measurement (Figure 1(b)). The DLS data showed the average particle sizes of CDs to be ~9.7 nm. The zeta potential of CDs was measured and is shown in Figure 1(c). Zeta potential was determined to be 36.2 mV indicating the positive charge on the CDs. Capping of CDs was carried out using *β*-cyclodextrin (*β*-cd), lactose monohydrate (LMH), and sucrose (Suc). PL properties of the CDs after capping were studied.  Figure 2 shows the photoluminescence (PL) spectra of CDs capped with *β*-cyclodextrin (*β*-cd), lactose monohydrate (LMH), and sucrose (Suc) and excited at 370 nm. The emission spectrum clearly shows that there is decrease in the fluorescence intensity (quenching) after capping with *β*-cd, lactose monohydrate (LMH), and sucrose (Suc) without any shift in the value of *λ*_em_.

**Figure 3:**
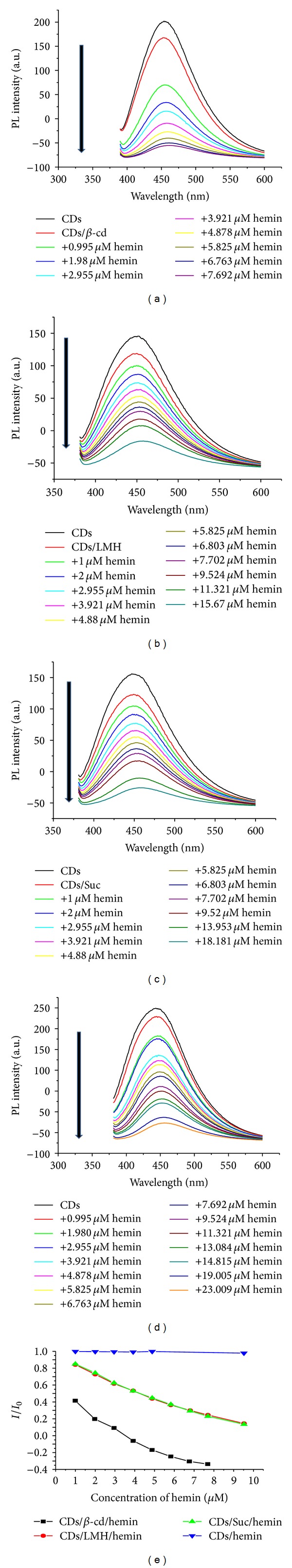
Photoluminescence emission spectra of (a) CDs/*β*-cd, (b) CDs/LHM, (c) CDs/Suc, and (d) bare CDs at different concentrations of hemin. (e) Plots of the donor fluorescence intensity against the concentration of hemin.

*β*-Cyclodextrin (*β*-cd), lactose monohydrate (LMH), and sucrose (Suc) capped carbon dots, namely, CDs/*β*-cd, CDs/LMH, and CDs/Suc systems, were then successfully used for sensing of hemin.  Figure 3(a) shows the stacked photoluminescence (PL) spectra of CDs/*β*-cd upon addition of different concentrations of hemin. It is evident from the spectrum that addition of hemin results in gradual decrease in emission intensity studied in the concentration range ~1 *μ*M−7 *μ*M after which the fluorescence intensity becomes almost constant. Similar behaviour in fluorescence intensity is observed for CDs/LMH and CDs/Suc systems.

Figure 3(b) shows the stacked photoluminescence (PL) spectra of CDs/LMH upon addition of hemin in the concentration range ~1 *μ*M–15.7 *μ*M after which the fluorescence intensity becomes constant. Similarly Figure 3(c) shows the stacked photoluminescence PL emission spectra of CDs/Suc upon successive addition of hemin in the concentration range ~1 *μ*M–18.18 *μ*M after which the fluorescence intensity becomes constant. The change in PL properties of bare carbon dots upon addition of hemin was also studied.  Figure 3(d) shows the stacked photoluminescence PL emission spectra of bare CDs on addition of hemin. In this case too the fluorescence intensity decreases in the concentration range ~1 *μ*M–23 *μ*M after which the fluorescence intensity becomes constant. The fact that CDs/*β*-cd shows more PL quenching towards detection of hemin than CDs/LMH and CDs/Suc is reflected in the plot of *I*/*I*_0_ versus concentration shown in Figure 3(e). The plot clearly shows that the detection of hemin as a result of net decrease in PL intensity is more pronounced with the CDs/*β*-cd capped system. The order of PL quenching from the plot was determined to be CDs/*β*-cd > CDs/LMH = CDs/Suc > CDs. Surprisingly CDs/LMH and CDs/Suc showed the same PL quenching towards hemin.

**Figure 4:**
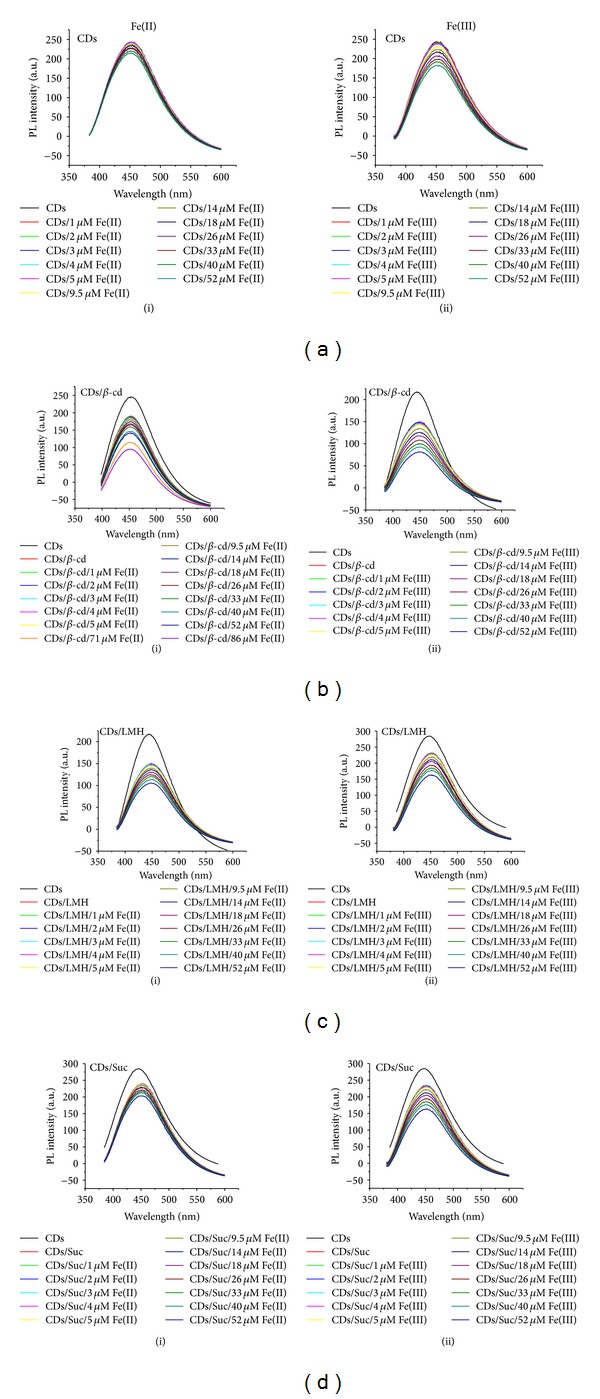
Photoluminescence emission spectra of (a) CDs at different concentrations of (i) free Fe(II) and (ii) free Fe(III), (b) CDs/*β*-cd at different concentrations of (i) free Fe(II) and (ii) free Fe(III), (c) CDs/LMH at different concentrations of (i) free Fe(II) and (ii) free Fe(III), and (d) CDs/Suc at different concentrations of (i) free Fe(II) and (ii) free Fe(III).

**Figure 5:**
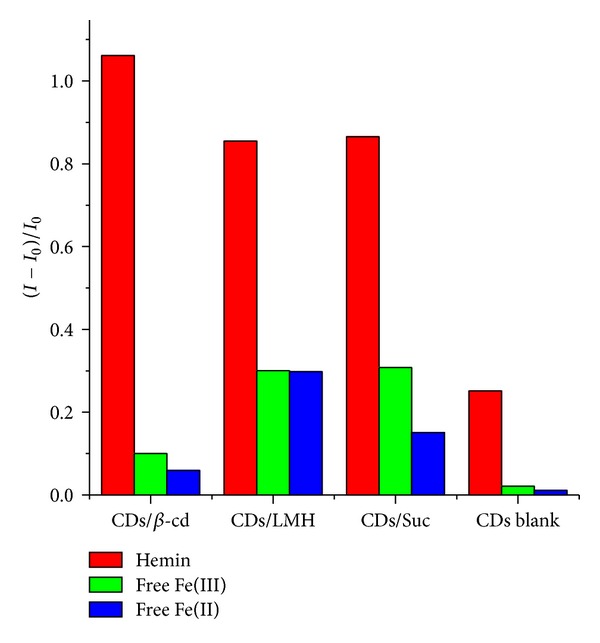
Comparative plot of change in PL intensity of carbon dots and carbon dot capped systems with regard to hemin, free Fe(II), and free Fe(III).

Detection of free Fe(III) and Fe(II) was also studied through the fluorescence emission properties of CDs/*β*-cd, CDs/LMH, and CDs/Suc.  Figure 4 shows the PL emission properties of CDs, CDs/*β*-cd, CDs/LMH, and CDs/Suc in the presence of free Fe(II) and Fe(III). The concentration range studied was from 1 *μ*m to 52 *μ*m. Overall in all the cases studied there was quenching of PL intensity of CDs, CDs/*β*-cd, CDs/LMH, and CDs/Suc upon addition of Fe(II) and Fe(III).  Figure 5 shows the comparison plot of the change in PL intensity with addition of iron, that is, Fe(III) in hemin, and free Fe(III) and Fe(II) into different systems, namely, CDs/*β*-cd, CDs/LMH, CDs/Suc, and bare CDs. The comparative plot points out the net PL quenching of different capped carbon dot systems towards hemin, free Fe(II), and free Fe(III). While CDs/LMH and CDs/Suc showed maximum quenching with free Fe(II) and Fe(III), bare carbon dots show least quenching with Fe(II) and Fe(III).

It is interesting to note that PL quenching which leads to detection of hemin is dependent on the capping agent. As discussed earlier the order of PL quenching in the presence of hemin is determined to be CDs/*β*-cd > CDs/LMH = CDs/Suc > CDs. Investigation of the structures of the capping agent reveals that net quenching is dependent on the number of –OH groups. Bare carbon dots (CDs) are prepared from chitosan so CDs are nothing but chitosan nanoparticles. Hence, CDs have three numbers of –OH groups per particle, LMH and Suc have eight numbers of –OH groups per molecule, and *β*-cd has a maximum of 21 numbers of –OH groups per molecule.

The system chosen for highly sensitive detection of hemin in aqueous media is fluorescent carbon dots prepared from chitosan hydrogel and capped with different hydroxyl groups bearing macromolecules like *β*-cyclodextrin and disaccharides like lactose monohydrate and sucrose. Capping of the CDs resulted in changes in their emission properties. The capped carbon dot systems, namely, CDs/*β*-cd, CDs/LMH, and CDs/Suc, show decreased PL intensity in the presence of hemin. The schematic representation illustrating the possible interactions involved in the detection of hemin by PL quenching is shown in Scheme 1(a). The observed PL emission in CDs is due to photogenerated recombination of electron-hole pairs, on the surface of the CDs as shown in Scheme 1(b). In our case when the surface of CDs is modified with the functional moiety it results in electron transfer (ET) between carbon dots and *β*-cd or lactose monohydrate or sucrose resulting in emission quenching. Among the various quenching mechanisms are inner filter effects, nonradiative recombination pathways, and electron transfer processes. Hemin has a pentacoordinate square pyramidal geometry with a d-orbital energy profile as shown in Scheme 1(b). The interaction of the –OH functional groups of the capping agent with the Fe(III) centre of hemin results in an octahedral symmetry around the Fe(III) atom which subsequently lowers the energy and raises the degeneracy of the energy levels. This provides increased stability to the complex and acts as the driving force for complex formation. Further, this lowering of energy level facilitates the electron transfer from the CDs/*β*-cd or CDs/LMH or CDs/Suc to hemin resulting in further quenching of PL intensity.

Moreover, the role of interaction of –OH group in the detection of hemin was also established by UV-Visible spectrophotometry. From the electronic absorption spectrum, it can be inferred that hemin in the pure state exhibits characteristic absorption bands at 612 nm, 385 nm, and 260 nm. The low energy band observed at 612 nm can be assigned to charge transfer transitions from the unsaturated porphyrin ligand system to the Fe(III) ion [19]. The intermediate energy band observed at 385 nm can be attributed to *π*-π* transition in the hemin complex. The high energy band observed at 260 nm can be attributed to *σ*-σ* transition in the hemin complex. On the other hand after interaction of the CDs/*β*-cd system with hemin the characteristic charge transfer band of hemin at 612 nm was shifted to longer wavelength and was found to appear at around 649 nm. This observation can be explained on the basis of the fact that the CDs/*β*-cd system has surface –OH groups which are electron donating groups and these on interaction with the Fe(III) centre in hemin further facilitate the charge transfer causing it to occur at lower energy and therefore at longer wavelength [20]. This observation provides a clear experimental evidence of the successful interaction of the surface –OH groups of the CDs/*β*-cd system with the Fe(III) centre in hemin.

## 4. Conclusion

In this work we successfully demonstrated the potential use of capped carbon dot systems, namely, CDs/*β*-cd, CDs/LMH, and CDs/Suc, as fluorescent sensors for detection of hemin. The capped carbon dot systems show quenching of PL intensity in the presence of hemin. The PL response of the capped systems with free Fe(II) and Fe(III) was also studied and found to be less sensitive than hemin. It is also an interesting finding that the net PL quenching which leads to detection of hemin is dependent on the number of –OH groups in the capping agent. The order of PL quenching towards hemin is determined to be CDs/*β*-cd > CDs/LMH = CDs/Suc > CDs. The reason is the interaction of –OH with hemin. Hemin molecule with pentacoordinate square pyramidal geometry can interact with –OH group of capping agent resulting in octahedral symmetry around the Fe(III) atom which subsequently lowers the energy and raises the degeneracy of the energy levels. This lowering of energy levels facilitates the electron transfer from the capped carbon dots to hemin resulting in further quenching of PL intensity. Such systems therefore have potential applications as biosensors.

## Figures and Tables

**Scheme 1:**